# Fast Proton Exchange in Histidine: Measurement of Rate Constants through Indirect Detection by NMR Spectroscopy

**DOI:** 10.1002/chem.201304992

**Published:** 2014-04-09

**Authors:** Akansha Ashvani Sehgal, Luminita Duma, Geoffrey Bodenhausen, Philippe Pelupessy

**Affiliations:** [a] Ecole Normale Supérieure, Département de Chimie, 24 rue Lhomond75231 Paris Cedex 05 (France), Fax: (+33) 1-44-32-24-02; [b] Université Pierre et Marie CurieParis (France); [c] UMR 7203, CNRS, Paris (France); [d] Ecole Polytechnique Fédérale de Lausanne, Institut des Sciences et d'Ingénierie ChimiquesBatochime, 1015 Lausanne (Switzerland)

**Keywords:** amino acids, exchange rate constants, NMR spectroscopy, protonation

## Abstract

Owing to its imidazole side chain, histidine participates in various processes such as enzyme catalysis, pH regulation, metal binding, and phosphorylation. The determination of exchange rates of labile protons for such a system is important for understanding its functions. However, these rates are too fast to be measured directly in an aqueous solution by using NMR spectroscopy. We have obtained the exchange rates of the NH_3_^+^ amino protons and the labile NH^ε2^ and NH^δ1^ protons of the imidazole ring by indirect detection through nitrogen-15 as a function of temperature (272 K<*T*<293 K) and pH (1.3<pH<4.9) of uniformly nitrogen-15- and carbon-13-labeled l-histidine**⋅**HCl**⋅**H_2_O. Exchange rates up to 8.5×10^4^ s^−1^ could be determined (i.e., lifetimes as short as 12 μs). The three chemical shifts *δ*_Hi_ of the invisible exchanging protons H_i_ and the three one-bond scalar coupling constants ^1^*J*(N,H_i_) could also be determined accurately.

## Introduction

Histidine is an essential amino acid that is often found in active sites of enzymes and other proteins that are involved in catalysis,[1] pH regulation,[2] metal binding,[3] and phosphorylation.[4], [5] The unique properties of histidine arise from its acid–base characteristics. Indeed, it is the only amino acid with an imidazole side chain that can act either as an acid or as a base in the physiological pH range. This property is of considerable significance for the function of many proteins. For example, NMR spectroscopy confirmed the catalytic role of His-12 and His-119 in RNase A,[6] the regulatory function of His-146 in human hemoglobin,[7] and the role of His-37 in shuttling protons into the virion by imidazole protonation and deprotonation, which is facilitated by ring reorientation.[8] Pioneering NMR spectroscopic studies based on direct proton detection have shown that the H^ε2^ and H^δ1^ protons of the imidazole ring exhibit slow exchange rates in RNase[9] and in human carbonic anhydrate.[10] Whereas NMR spectroscopy has been used to study ^15^N chemical shifts and ^2^*J*(N,C) and ^3^*J*(N,C) in the imidazole rings of histidine over a wide pH range,[11,12] to the best of our knowledge, the exchange rates of labile protons of histidine in aqueous solution have not yet been reported. Solid-state NMR spectroscopy has been used to study ^15^N and ^13^C chemical shifts of histidine in single crystals and in lyophilized and microcrystalline powders,[13–16] whereas isotropic and anisotropic ^15^N chemical shifts were exploited to investigate the tautomeric and acid–base equilibria of histidine.[13,14] A linear correlation was found between the isotropic ^15^N chemical shifts in imidazole and the degree of bond stretching induced by hydrogen bonding.[17] Interestingly, the ^13^C chemical shifts of imidazole in histidine lyophilized from solutions with various pH values conserved information about the p*K*_a_ values of the parent solutions.[15]

Although NMR spectroscopy is unparalleled for the determination of exchange rates owing to its capacity to provide site-specific information, its scope is normally limited to moderately fast exchange rates (up to few thousand s^−1^ under favorable conditions).[18,19] Recently, we were able to extend the range of exchange rates that can be measured by NMR spectroscopy by more than an order of magnitude (up to 10^5^ s^−1^ for an NH group) by using a method that accurately determines the effects on ^15^N nuclei of scalar relaxation due to exchanging protons.[20] This method relies on monitoring the decay of ^15^N magnetization under multiple refocusing Carr–Purcell–Meiboom–Gill (CPMG)[21,22] trains in the presence or absence of proton decoupling. In this work, we use this approach to measure fast-exchange rate constants of the H^δ1^ and H^ε2^ protons of the imidazole ring and of the NH_3_^+^ protons in histidine as a function of pH and temperature. Furthermore, we determined the elusive one-bond scalar coupling constants ^1^*J*(N,H_i_) and chemical shifts *ω*_Hi_ of the otherwise invisible H^δ1^, H^ε2^, and NH_3_^+^ protons.

### Methodology

#### Pulse sequence

**Figure 1:**
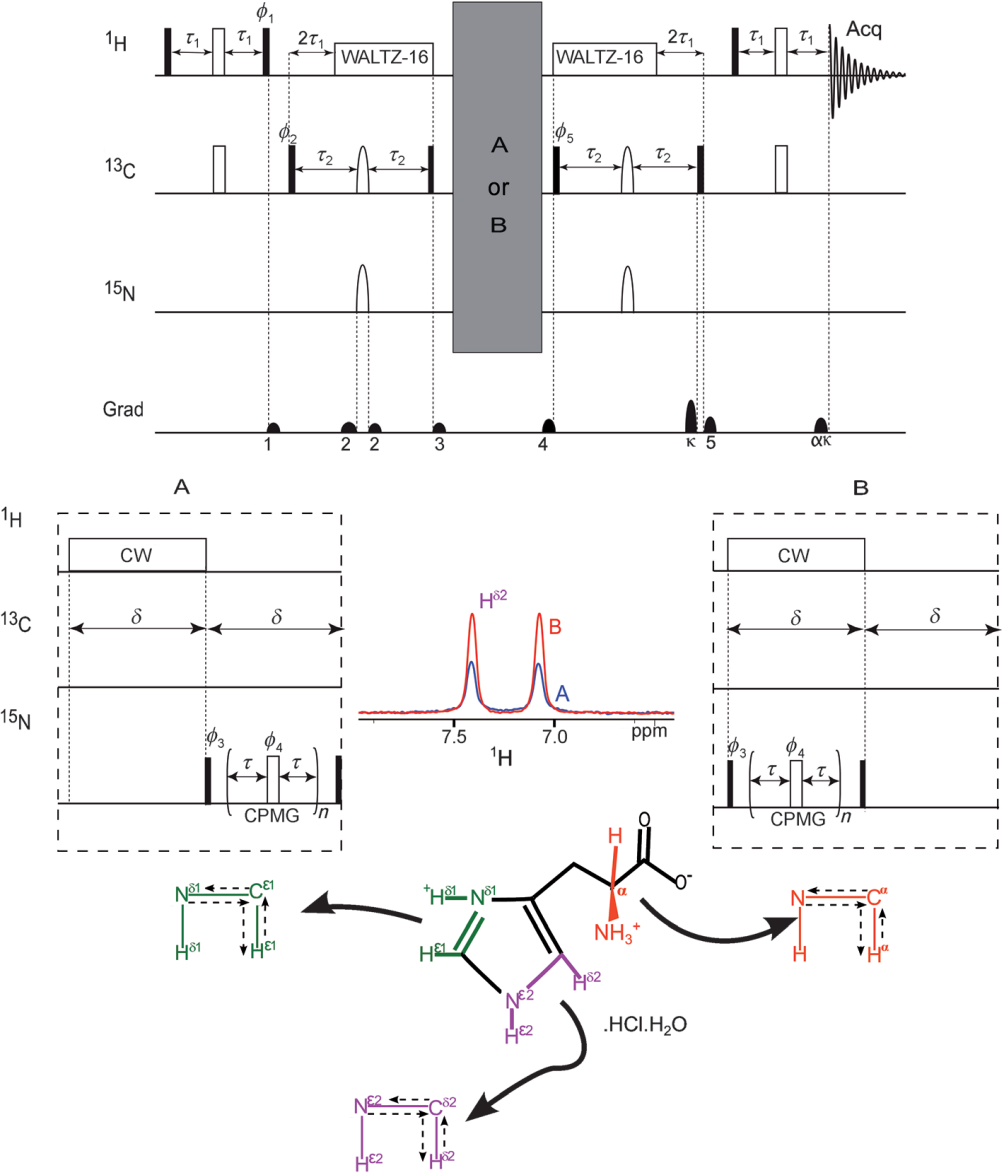
Pulse sequence designed to measure the proton exchange rates *k*_ex_ of the NH^δ1^, NH^ε2^, and NH_3_^+^ groups in histidine. The *π* and *π*/2 pulses are represented by narrow open and filled rectangles, respectively, whereas decoupling sequences are represented by wide rectangles. Continuous-wave (cw) proton decoupling was applied in both experiments A and B for a duration *δ*, but the CPMG train, also of length *δ*, was delayed until after the decoupling interval in experiment A, whereas both were applied at the same time in experiment B. This prevents differences in temperature induced by decoupling. All *π*/2 and *π* pulses applied to ^13^C in the first and last INEPT blocks were nonselective rectangular pulses, whereas the ^13^C refocusing pulses in the other INEPT blocks had REBURP profiles[35] with a duration of 4 ms. The ^15^N inversion *π* pulse in the INEPT block had a REBURP profile of 2 ms duration for observing NH_3_^+^ protons and Q3 profiles[36] with a duration of 30 ms for probing the NH^ε2^ and NH^δ1^ protons. Continuous-wave proton decoupling was used during the blocks A and B but WALTZ-16 decoupling[37] was used during the INEPT sequences that bring about coherence transfer between ^13^C and ^15^N. The delays were set to *τ_1_*=1.69 ms≈1/(4 ^1^*J*(C,H)), *τ*_2_=23.43 ms≈1/(4 ^1^*J*(N,C)) for NH_3_^+^, *τ*_1_=1.22 ms≈1/(4 ^1^*J*(C,H)), *τ*_2_=16.7 ms≈1/(4 ^1^*J*(N,C)) for NH^ε2^, *τ*_1_=1.12 ms≈1/(4 ^1^*J*(C,H)), *τ*_2_=17.9 ms≈1/(4 ^1^*J*(N,C)) for NH^δ1^. All phases were along the *x* axes unless indicated otherwise. The phases were cycled according to: *ϕ*_1_=2{*y*}, 2{−*y*}, *ϕ*_2_={*x*}, {−*x*}, *ϕ*_3_=4{*x*}, 4{−*x*}, *ϕ*_4=_*y*, *ϕ*_5_=8{*x*}, 8{−*x*} with a receiver phase *ϕ*_rec_={*x*, −*x*, −*x*, *x*, −*x*, *x*, *x*, −*x*, −*x*, *x*, *x*, −*x*, *x*, −*x*, −*x*, *x*}. The gradients need to be carefully adjusted to avoid accidental refocusing. The value *α*=*γ*_C_/*γ*_H_. The labile H^δ1^, H^ε2^, and NH_3_^+^ protons examined in this work are highlighted with colors and the pathways for the transfer of magnetization are indicated on the molecular structure. By way of example, spectra A and B of the H^δ2^ proton are shown for indirect detection of NH^ε2^ at pH 3.2 and 292.5 K.

The pulse sequences designed to determine the exchange rate constants of the labile H^ε2^, H^δ1^, or NH_3_^+^ protons of histidine are shown in Figure 1. The magnetization of a neighboring ‘spy’ proton is transferred via the adjacent carbon-13 to the target nitrogen-15 in the form of antiphase coherences 2 N^ε2^_*y*_C^δ2^_*z*_, 2 N^δ1^_*y*_C^ε1^_*z*_, or 2 N_*y*_C^α^_*z*_ by means of two successive coherence transfer steps in the manner of insensitive nuclei enhanced by polarization transfer (INEPT).[23] These antiphase N-spin coherences are then allowed to decay under a multiple-refocusing CPMG pulse train applied to the ^15^N nuclei. In experiment B of Figure 1, continuous wave (cw) proton decoupling is applied during the ^15^N pulse train, whereas in experiment A of Figure 1, decoupling is only applied before the pulse train when the nitrogen magnetization is still along the *z* axis. The relaxation of the C^δ2^_*z*_, C^ε1^_*z*_, and C^α^_*z*_ terms equally affects experiments A and B and does not play any role in subsequent analyses. The remaining antiphase coherences are then transferred back to the spy protons. Experiments have been recorded for temperatures between 272.6 and 292.5 K. At each temperature and for each ^15^N nucleus, about 20 different sets of experiments were performed with variable numbers 2<*n*<32 of *π* pulses in the CPMG pulse trains, different pulse intervals (*τ*=1.25, 2.5, 5 ms), different offsets of the radio frequency (*rf*) carrier (varied in steps of 2 kHz), and different amplitudes of the cw proton decoupling field (from 156 to 5000 Hz). Each set of experiments comprised four consecutive pairs of experiments A and B and typically lasted about 3 min. From the ratios *A*/*B* of the signal intensities observed in experiments A (without proton decoupling during the ^15^N CPMG train) and B (with proton decoupling), the rate constants of the proton exchange were calculated using a home-written Mathematica program. A small correction was applied to account for the presence of 3 % D_2_O in the solvent.[20]

#### Theoretical treatment

Proton exchange can be mediated by H_2_O, OH^−^, or H_3_O^+^. If the incoming and outgoing protons have opposite polarization, this leads to a flip-flop of the spin attached to the nitrogen. From the point of view of a ^15^N coherence, this amounts to an interconversion of the two lines of the doublet, that is, an exchange of the two single transition operators [Eq. (1)]:

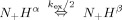
(1)

Since a proton exchange leads only to a 50 % likelihood of a spin flip, the exchange rate is divided by two. In a base of Cartesian product operators, the exchange leaves the in-phase operator *N_x_* invariant, and it contributes to the auto-relaxation rate of the antiphase operator 2 *N_y_H_z_* since a spin flip leads to [Eq. (2)]:

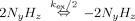
(2)

The exchange constants can be obtained by solving the Liouville–von Neumann equation [Eq. (3)] during the CPMG pulse train:

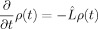
(3)

in which *ρ*(*t*) denotes the density operator and 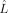 represents the Liouvillian superoperator,[24] which includes coherent evolution, relaxation, and exchange contributions.[25] The solution of this first-order differential equation describes the evolution of the density operator during the CPMG pulse train that contains *n π* pulses spaced by intervals 2*τ* [Eq. (4)]:

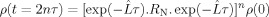
(4)

in which *R*_N_ represents the *π_y_* pulse applied to ^15^N. In the basis {*N_y_*, 2 *N_x_H_z_*, 2 *N_x_H_y_*, 2 *N_x_H_x_*}, this leads to [Eqs. (5) and (6)]:

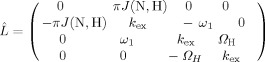
(5)

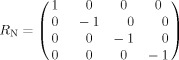
(6)

Here *ω*_1_ is the amplitude of the rf decoupling field applied to the protons, *J*(N,H) is the relevant one-bond scalar coupling constant, and *Ω*_H_ is the offset between the chemical shift *ω*_H_ of the exchanging proton (H^δ1^, H^ε2^, or NH_3_^+^) and the carrier frequency *ω*_rf_ [Eq. (7)]:

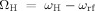
(7)

The ratio *A*/*B* depends on the offset since the decoupling becomes less efficient when the proton carrier frequency is off-resonance.[20] Thus for a given number of pulses and a given duration of the interpulse delay, the ratio is *A*/*B* smallest when the proton carrier coincides with the chemical shift of the exchanging proton.

We have considered the pulses applied to the ^15^N nuclei to be instantaneous (in reality they are about 70 μs long, much shorter than the shortest interval of 2*τ*=2.5 ms). In Equation (5), relaxation is not taken into consideration since the rates either affect both experiments A and B equally or can be neglected relative to the exchange rates. To obtain the rate constant, the density operator is calculated twice to get the ratios *A*/*B* [Eq. (8)]:

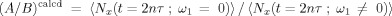
(8)

In this treatment, we considered the exchanging protons to belong to NH groups; NH_2_ or NH_3_ groups can be treated like an NH group except that one has to take the square or cubic root, respectively, of the experimental *A*/*B* ratios.[26]

**Figure 2:**
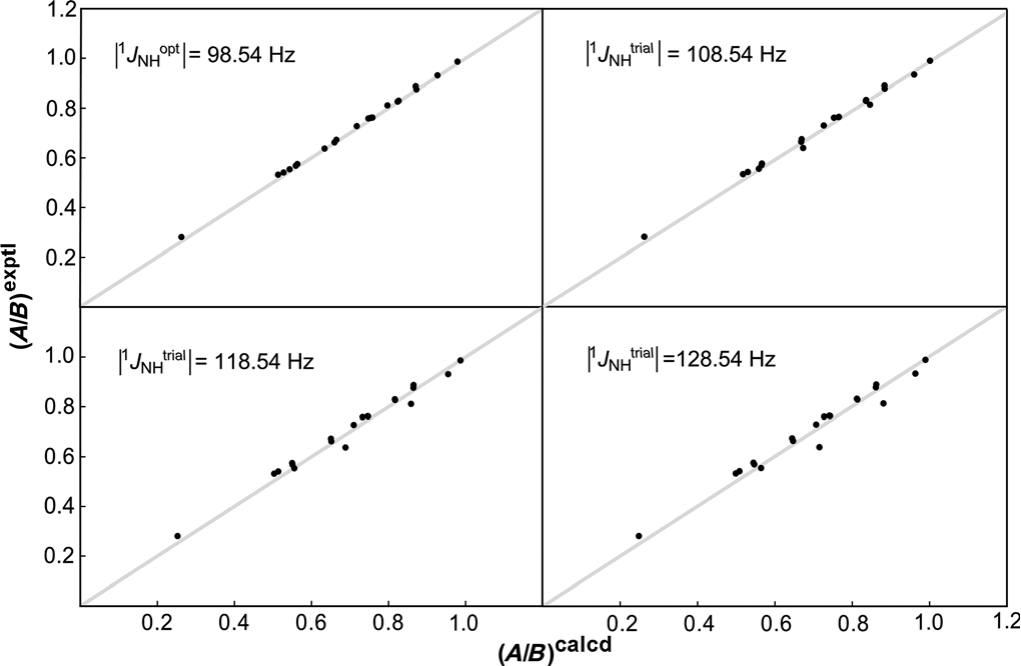
The scalar couplings ^1^*J*(N,H) can be postulated to have values ^1^*J*(N,H)^trial^ that differ from the optimum value |^1^*J*(N,H)^opt^|=98.54 Hz, whereas the chemical shift *δ*_H_ and exchange rate *k*_ex_ of the H^ε2^ proton at pH 3.2 and 292.5 K are fitted. Significant discrepancies are observed when the scalar coupling is deliberately miss-set to |^1^*J*(N,H)^trial^*|*−|^1^*J*(N,H)^opt^|=0, 10, 20, or 30 Hz. The gray line with a unit slope is merely to guide the eye. Similar results have been obtained for different sites, pH values, and temperatures.

**Figure 3:**
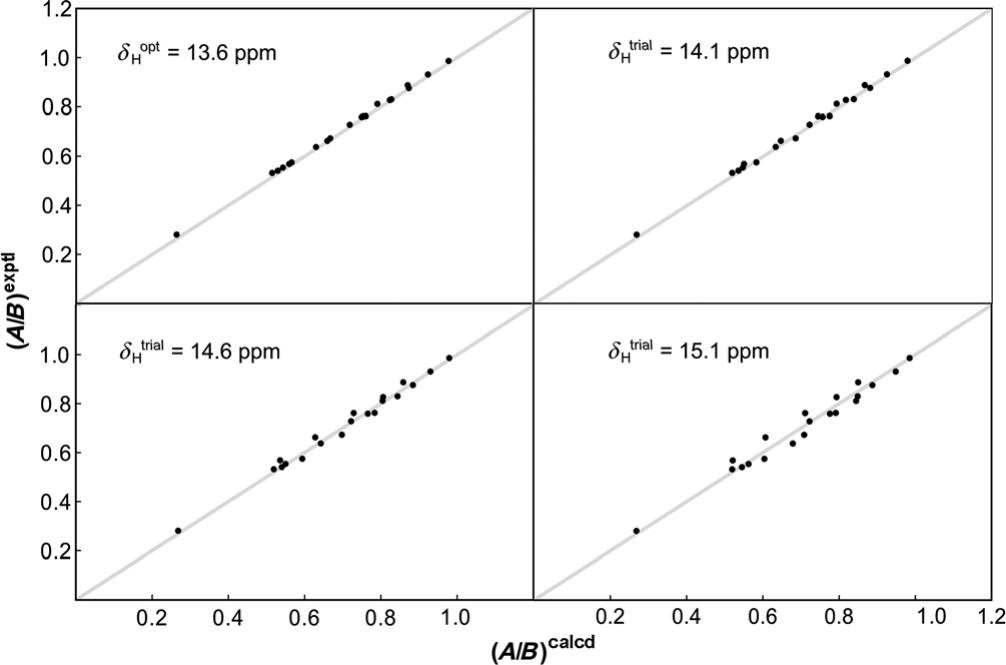
The chemical shifts *δ*_H_ of the exchanging NH^ε2^ proton can be miss-set deliberately to values *δ*_H_^trial^ that differ from the optimum *δ*_H_^opt^=13.6 ppm while fitting the one-bond scalar coupling constant ^1^*J*(N,H) and the exchange rate *k*_ex_ at pH 3.2 and 292.5 K. Significant discrepancies are observed when the chemical shift is deliberately miss-set to *δ*_H_^trial^−*δ*_H_^opt^=0, 0.5, 1, or 1.5 ppm. The experimental ratios (*A*/*B*)^exptl^ are plotted against the calculated ratios (*A*/*B*)^calcd^. The gray line with a unit slope is to guide the eye. Similar results have been obtained for different sites, pH values, and temperatures.

In our previous work, the exchange rate could in principle be determined from only two ratios *A*/*B* obtained with two different intervals 2*τ* since the resonance frequencies *Ω*_H_ and scalar couplings *J*(N,H) were known precisely. Under these conditions, the Liouvillian of Equation (5) has only one unknown, but two different experiments are nevertheless necessary since there are two degenerate solutions.[20,26] In this work, the labile protons remain invisible over the entire pH range because they exchange too rapidly. Hence, *n*, *τ*, *ω*_rf_, and *ω*_1_ were varied systematically. For all experimental conditions, the ratio (*A*/*B*)^calcd^ of Equation (8) was determined using a home-written Mathematica program. Then the values of *ω*_H_, *J*(N,H), and *k*_ex_ were obtained by minimizing *χ*^2^, the sum of the squares of the deviations (*A*/*B*)^exptl^−(*A*/*B*)^calcd^. Figures 2 and 3 show that the experiments allow one to accurately determine all three unknown parameters *k*_ex_, *J*(N,H), and *δ*_H_: when only two of the unknown parameters are fitted, whereas the third one is purposely miss-set to *J*(N,H)^test^ or *δ*_H_^test^, significant deviations between the calculated and experimental ratios appear. The procedure described above does not allow one to obtain meaningful exchange rates when the *A*/*B* ratio is close to 1. When 0.9<*A*/*B*<1.0 for an entire set of experiments at a given pH and temperature, the fits become rather imprecise and sensitive to small systematic deviations. Hence, we only applied the three-parameter fit for pH values and temperatures for which *A*/*B*<0.9 in at least one of the experiments recorded at a given pH and temperature. From these fits we obtained a scalar coupling constant *J*(N,H) and a chemical shift *ω*_H_. It is known that ^1^*J*(^15^N,^1^H)<0,[27] but our results do not depend on the sign of the scalar coupling, hence only its absolute value can be deduced. Then the whole set of experiments was analyzed again by assuming that the scalar coupling constants and chemical shifts are constant over the entire pH range. Thus, the exchange rates *k*_ex_ of Figure 6 (see below) were determined by fitting only a single parameter (i.e., the exchange rate).

**Figure 4:**
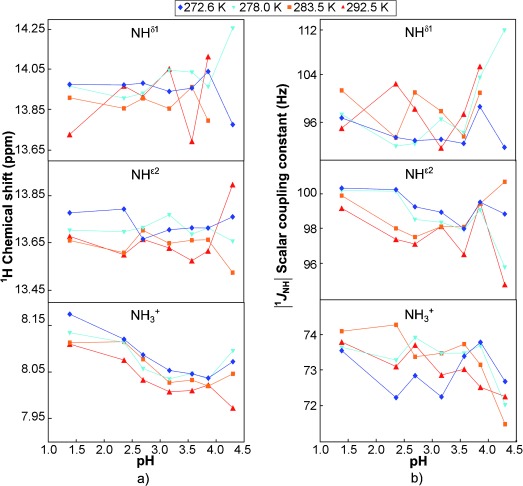
Variations of a) the chemical shifts *δ*_H_ and b) scalar coupling constants ^1^*J*(N,H) that have been determined over a range 1.0<pH<4.5 and temperatures 272.6<*T*<292.5 K, encoded by different colors. The chemical shifts *δ*_H_^opt^ were referenced with respect to the temperature-dependent shift *δ*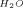 of the water resonance, as determined by Markley et al.[38] The variations are very small (on the order of 0.1 ppm). For the NH_3_^+^ group, the chemical shift appears to have a linear dependence on pH. However, whether one simply assumes an average shift <*δ*_H_> or a linear pH dependence of the chemical shifts, the resulting exchange rates are not significantly affected.

The scalar coupling constants determined in this fashion are |^1^*J*(N,H_3_^+^)|=(73.2±0.2) Hz, |^1^*J*(N,H^ε2^)|=(98.5±0.3) Hz, and |^1^*J*(N,H^δ1^)|=(97.2±1.0) Hz. The stochastic errors in the *A*/*B* ratios owing to noise were very small (on the order of 1 ‰). Consequently, systematic errors due to rf miscalibration or inhomogeneities, small temperature variations, instrumental instabilities and theoretical simplifications (in particular, cross-correlated relaxation effects were neglected) are expected to be dominant. Hence all *A*/*B* ratios were weighed equally in the *χ*^2^ minimization. The errors in the scalar coupling constants and chemical shifts were determined from standard deviations of different experiments. In Figure 4, the chemical shifts of the different NH protons are plotted as a function of pH.

To determine the exchange rates, we averaged the scalar coupling constants *J*(N,H) over all temperatures and pH values, whereas we considered the chemical shifts separately for each temperature and only averaged them over all pH values. The ^1^H chemical shift of the NH_3_^+^ group seems to depend slightly on pH; however, the fit of the exchange rate is not very sensitive to such small variations, which are on the order of *δ*=0.1 ppm. Systematic errors in the exchange rates are difficult to assess, whereas stochastic errors are very small. Therefore, we assumed the error to be the square root of the sum of two sources: 1) errors of 1 % common to all rates, which correspond to an rf miscalibration of about 1 %, and 2) errors in the fits owing to systematic deviations of 1 ‰ of the *A*/*B* ratios. Additionally, there is a contribution due to the error in the scalar coupling. For a given *A*/*B* ratio, the exchange rate is (to a good approximation) proportional to the square of the scalar coupling, which leads to errors of 0.5, 0.8, and 2 % for NH_3_^+^, NH^ε2^, and NH^δ1^, respectively. The latter errors lead to a synchronous shift of all rates of a particular proton and should therefore be considered separately.

In our analysis, we neglected modulations of the isotropic chemical shifts and scalar couplings induced by de- and reprotonation. Modulations of the nitrogen shifts affect experiments A and B equally and do not affect the *A*/*B* ratios, although such modulations can lead to line broadening and hence to a decrease in the signal-to-noise ratio. In principle, modulations of the isotropic proton shifts and scalar couplings can affect the *A*/*B* ratios. However, we found an excellent agreement between calculations and experimental values by using a single value for these parameters. These values might need to be interpreted as weighted averages over different conformers.

## Results and Discussion

The protons of the NH^δ1^, NH^ε2^, and NH_3_^+^ systems have distinct chemical shifts (*δ*_H_), scalar coupling constants (*J*(N,H)), and exchange rates (*k*_ex_), but none of these can be observed directly in proton spectra because of fast exchange. We have measured the exchange rate constants of the labile H^δ1^, H^ε2^, and NH_3_^+^ protons in histidine over a pH range from 1.39 to 4.88 (Figure 6, see below). This range is well below the p*K*_a_ values of the NH^δ1^ group of the imidazole ring (p*K*_a_=6.0) and of the NH_3_^+^ group (p*K*_a_=9.17) so that the nitrogen atoms N^ε2^ and N^δ1^ of the imidazole ring and the amino group NH_3_^+^ have protonation fractions close to 1. Since histidine is in a fully protonated state over this pH range, the propensity of exchange mediated by hydronium ions is expected to be small.[28] The exchange rate constants that could be determined range from *k*=2.5×10^2^ to 8.5×10^4^ s^−1^. For the exchange rates of the NH^δ1^ and NH^ε2^ groups, we observed a plateau up to pH 3 and thereafter a gradual increase with pH. This happens because the exchange is water-mediated and obeys pseudo-first-order kinetics at low pH but becomes base-mediated with increasing pH.[28] For amino acids, exchange through base catalysis is far more effective than acid catalysis by as much as eight orders of magnitude.[29] For NH_3_^+^, we found a U-shaped curve when plotting the exchange rate on a logarithmic scale versus pH. A small contribution of acid catalysis allows one to explain this shape (see below).

In our previous work on the indole protons of tryptophan, we were able to measure exchange rates up to 10^5^ s^−1^.[20] The p*K*_a_ values of tryptophan span a wide range from 2.38 for the carboxyl to 9.39 for the amino group. The catalysis of proton exchange in tryptophan occurs mainly through H^+^ and OH^−^ ions to give a well-defined V-shaped curve of log (*k*_ex_) versus pH, a feature that is not found in histidine. The minimum of the curve at about pH 4–5 is due to the electron-withdrawing inductive effect of the indole side chain.[30]

**Figure 5:**
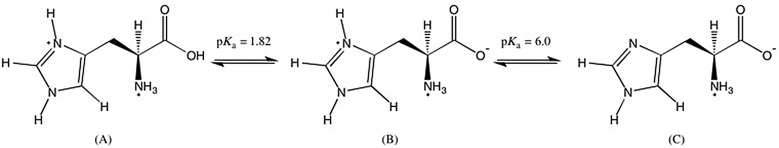
In the range 1<pH<5, histidine is mainly present in three forms (A, B, C). The p*K*_a_ of the equilibrium between A and B is 1.82, whereas the p*K*_a_=6.0 for the equilibrium between B and C.

In histidine, for the pH range studied here, the rate constants are averaged over three distinct forms in solution (Figure 5), the relative concentrations of which are described by the following fractions [Eq. (9)]:

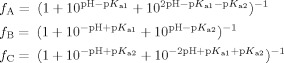
(9)

**Figure 6:**
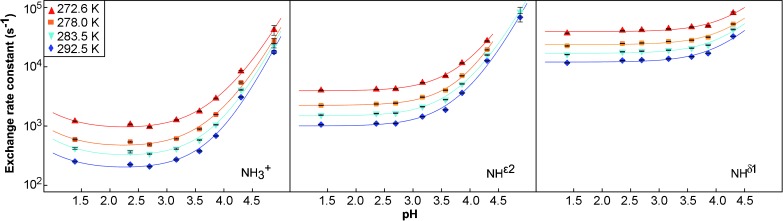
Proton-exchange rate constants *k*_ex_ obtained for the NH_3_^+^, NH^ε2^, and NH^δ1^ groups in histidine as a function of 1.0<pH<4.9 over the temperature range 272.6<*T*<292.5 K. Most error bars are smaller than the symbols. Errors due to the uncertainty of the scalar couplings are not comprised in the error bars and cause a systematic shift of all points, as explained in the text. The solid lines result from fits to Equations (9) and (10).

The p*K*_a1_=1.82 refers to the carboxyl group, and p*K*_a2_=6.0 corresponds to the H^δ1^ proton of the imidazolium ring.[31] Each of the three forms can have acidic and alkaline contributions to the exchange rates of the three labile H^δ1^, H^ε2^, and NH_3_^+^ protons and a common contribution *W* that stems from exchange with neutral water. The solid lines in Figure 6 were obtained by fitting the rate constants with the exchange equations [Eq. (10)]:

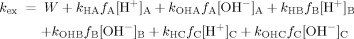
(10)

in which [H^+^]=10^−pH^, [OH^−^]=*K*_W_10^pH^, and *K*_W_ is the auto-ionization constant of water, which depends on the temperature.[32] The alkaline contribution (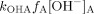) of form A and the acidic contribution (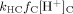) of form C can be neglected, since at pH values for which *f*_A_ and *f*_C_ are significant these contributions are eclipsed by other terms in Equation (10). For NH^δ1^, only two parameters (*W* and *k*_OHB_) were sufficient to explain the trend (the acid-mediated contributions appear negligible and the ring in the C form has no H^δ1^). For NH^ε2^, three parameters were needed (*W, k*_OHB_, and *k*_OHC_), whereas the acid-mediated rate constants *k*_HA_ and *k*_HB_ could not be determined. For NH_3_^+^, an additional acid-mediated contribution had to be included to explain the slight increase of the exchange rate at low pH. The same acid-mediated rate constant was assumed for both forms A and B (*k*_HA_=*k*_HB_), since the data do not allow for sufficient discrimination. All exchange constants that could be determined are shown in Table 1.

**Table 1:** Exchange rate constants of the protons in the NH_3_^+^, NH^ε2^, and NH^δ1^ groups of histidine.

		*T*[K]			
		272.6	278.0	283.5	292.5
NH_3_^+^	log (*W*/[m^−1^ s^−1^])	2.27	2.48	2.64	2.95
	log (*k*_HA_/[m^−1^ s^−1^])	3.21	3.47	3.60	3.90
	log (*k*_OHB_/[m^−1^ s^−1^])	13.69	13.68	13.69	13.62
	log (*k*_OHC_/[m^−1^ s^−1^])	15.44	15.30	15.11	14.87
NH^ε2^	log (*W*/[m^−1^ s^−1^])	3.01	3.17	3.35	3.60
	log (*k*_OHB_/[m^−1^ s^−1^])	14.43	14.38	14.24	14.16
	log (*k*_OHC_/[m^−1^ s^−1^])	15.95	15.79	15.86	15.43
NH^δ1^	log (*W*/[m^−1^ s^−1^])	4.08	4.23	4.38	4.59
	log (*k*_OHB_/[m^−1^ s^−1^])	14.94	14.83	14.69	14.51

**Figure 7:**
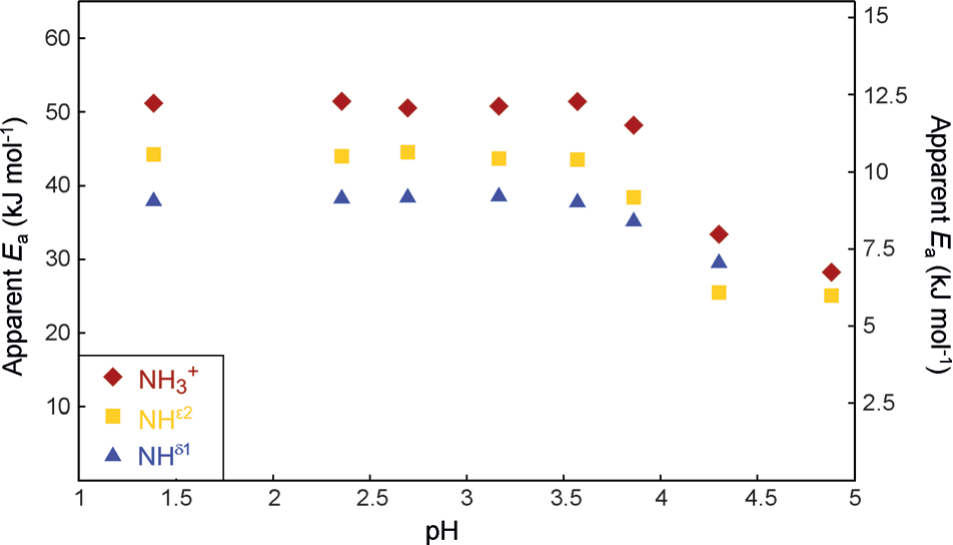
Apparent activation energies of proton-exchange processes in the NH_3_, NH^ε2^, and NH^δ1^ groups in histidine as a function of pH obtained from the derivatives of the logarithms of the exchange rates of Figure 6 with respect to 1/*T*.

The apparent activation energies of the three H^δ1^, H^ε2^, and NH_3_^+^ exchange processes were estimated from the temperature dependence of the proton exchange rates over the relevant range of pH values. As expected, the proton exchange rates increase with increasing temperature for all sites (Figure 6), and the activation energy can be determined for each NH group at each pH. The apparent activation energies are provided in Figure 7 as a function of pH. We assume that the pH does not depend on the temperature over the limited temperature range considered. The activation energy is roughly constant at low pH and then decreases with increasing pH. These apparent activation energies provide information about the barrier height and thus give insight into the strength of hydrogen bonds. The NH^δ1^, NH^ε2^, and NH_3_^+^ groups are involved in hydrogen bonds with partners (H_2_O or OH^−^) with which they can exchange a proton. The average chemical shifts of all exchanging protons is below *δ*=16 ppm. According to Hong et al.[33] this implies that the hydrogen bonds can be described by asymmetric energy wells with unequal populations. The elongation of hydrogen bonds plays an important role in exchange processes.[28,34] The stronger the hydrogen bond, the more energetically expensive its elongation, thus leading to a higher energy barrier and a slower exchange rate. As indicated in the Figure 7, the barrier is lowest for NH^δ1^, which suggests weak hydrogen bonds in the transition states that involve H_2_O or OH^−^ as partners, thereby facilitating exchange. This corroborates the correlation between the activation energy and the exchange rate. At a given pH and temperature (as shown in Figure 6), we obtained *k_ex_*(NH_3_^+^)<*k*_ex_(NH^ε2^)<*k*_ex_(NH^δ1^). The exchange rates of the three different groups are separated by about an order of magnitude.

Our method should also be applicable to measuring exchange rates that involve deuterium nuclei, and hence investigating isotope effects on these rates. The equations need to be adapted to take into account the quadrupolar nature of the deuterons that have spin quantum number *I*=1 so that the ^15^N spectrum features a triplet. Furthermore, the exchange rate for a given *A*/*B* ratio approximately scales with the square of the ratio ^1^*J*(N,D)/^1^*J*(N,H)=γ_D_/γ_H_≈0.15, so that the highest accessible rates will be much lower.

## Conclusion

We have presented a method for measuring the proton exchange rates *k*_ex_ of the NH^δ1^, NH^ε2^, and NH_3_^+^ groups in histidine without any prior knowledge of the proton chemical shifts *δ*_H_ and the one-bond scalar coupling constants ^1^*J*(N,H). Our approach permits one to determine these parameters indirectly. The three exchange rates were measured at different temperatures and pH values to determine the apparent activation energies of the exchange processes as a function of pH. The same methods can be applied to histidine residues embedded in proteins, provided the transverse relaxation rates of ^1^H, ^13^C, and ^15^N are not too fast. Altogether, these results provide valuable information about the role of pH in the chemistry of histidine.

## Experimental Section

All experiments were performed at 14 T (600 MHz for ^1^H) using a Bruker Avance III spectrometer equipped with a triple-channel indirect detection probe. Temperature calibration was performed using a mixture of protonated/deuterated (4:96) methanol.

### Sample preparation and pH measurements

U-[^13^C,^15^N]-labeled histidine**⋅**HCl**⋅**H_2_O was purchased from Cortecnet (France). 20 mm solutions of histidine with a pH that ranged from 1.39 to 4.88 were prepared using HCl (1 m, 0.1 m, 0.01 m) or NaOH (1 m, 0.1 m). No buffers were used in this work to avoid their contributions to the hydrogen exchange rates. The pH measurements were performed using a pH meter (Hach Langer) before and after the experiments, and the average was retained in the subsequent analysis. Since the experiments were performed without adding any buffer, new NMR spectroscopy tubes were scrupulously cleaned to prevent any pH drift by using a NMR spectroscopy tube cleaning apparatus from Sigma Aldrich. The tubes were filled with concentrated nitric acid and allowed to stand overnight and were subsequently rinsed copiously with water.
