# Supplementary material for: Fast Proton Exchange in Histidine: Measurement of Rate Constants through Indirect Detection by NMR Spectroscopy
Source: Chemistry. 2014 Apr 9;20(21):6332–8. doi: 10.1002/chem.201304992 (PMC4497323; doi:10.1002/chem.201304992)
Supplement: Supplementary file 1 [file chem0020-6332-sd1.pdf]

CHEMISTRY
A European Journal

Supporting Information

© Copyright Wiley-VCH Verlag GmbH & Co. KGaA, 69451 Weinheim, 2014

Fast Proton Exchange in Histidine: Measurement of Rate Constants through Indirect Detection by NMR Spectroscopy

Akansha Ashvani Sehgal,[a, b, c] Luminita Duma,[a, b, c] Geoffrey Bodenhausen,[a, b, c, d] and Philippe Pelupessy*[a, b, c]

chem_201304992_sm_miscellaneous_information.pdf

Table S1. Absolute values of one-bond scalar coupling constants for a) NH_3^+, b) $NH^{\varepsilon 2}$ and c) $NH^{\delta 1}$:

a) $|^1J(NH_3^+)|$ Hz				
**Average pH**	**272.6 K**	**278.0 K**	**283.5 K**	**292.5 K**
**1.39**	73.54	73.64	74.10	73.79
**2.36**	72.22	73.28	74.28	73.09
**2.70**	72.84	73.92	73.37	73.70
**3.17**	72.24	73.47	73.47	72.86
**3.57**	73.39	73.47	73.73	73.02
**3.86**	73.78	73.69	73.14	72.51
**4.30**	72.67	72.02	71.49	72.25
b) $|^1J(NH^{\varepsilon 2})|$ Hz				
**1.39**	100.34	100.20	99.90	99.16
**2.36**	100.25	100.16	97.99	97.36
**2.70**	99.24	98.51	97.49	97.09
**3.17**	98.93	98.34	98.10	98.11
**3.57**	97.97	97.90	98.07	96.50
**3.86**	99.51	99.07	99.42	99.48
**4.30**	98.82	95.79	100.71	94.81
c) $|^1J(NH^{\delta 1})|$ Hz				
**1.39**	96.71	97.29	101.25	95.06
**2.36**	93.61	92.27	93.58	102.39
**2.70**	93.13	92.66	100.91	98.19
**3.17**	93.31	96.51	97.80	91.99
**3.57**	92.67	94.38	93.77	97.33
**3.86**	98.54	103.49	100.86	105.40
**4.30**	92.08	111.99		

Table S2. Chemical shifts δ_H of a) NH_3^+ protons, b) $NH^{\varepsilon2}$ proton and c) $NH^{\delta1}$ proton:

**a) δ_H (NH_3^+) ppm**				
**Average pH**	**272.6 K**	**278.0 K**	**283.5 K**	**292.5 K**
**1.39**	8.174	8.134	8.113	8.109
**2.36**	8.120	8.113	8.114	8.075
**2.70**	8.086	8.057	8.077	8.032
**3.17**	8.052	8.034	8.027	8.007
**3.57**	8.045	8.046	8.032	8.009
**3.86**	8.036	8.033	8.019	8.021
**4.30**	8.071	8.095	8.045	7.972
**b) δ_H ($NH^{\varepsilon2}$) ppm**				
**1.39**	13.777	13.703	13.659	13.677
**2.36**	13.793	13.697	13.608	13.599
**2.70**	13.665	13.714	13.702	13.664
**3.17**	13.704	13.768	13.647	13.628
**3.57**	13.713	13.686	13.660	13.574
**3.86**	13.712	13.712	13.662	13.615
**4.30**	13.760	13.657	13.524	13.898
**c) δ_H ($NH^{\delta1}$) ppm**				
**1.39**	13.974	13.965	13.907	13.726
**2.36**	13.971	13.905	13.855	13.966
**2.70**	13.980	13.930	13.904	13.914
**3.17**	13.939	14.045	13.854	14.052
**3.57**	13.955	14.035	13.961	13.693
**3.86**	14.038	13.963	13.795	14.113
**4.30**	13.775	14.257		

Table S3. Exchange rate constants k_{ex} for a) NH_3^+ protons, b) $NH^{\varepsilon2}$ proton and c) $NH^{\delta1}$ proton:

**a) k_{ex} (NH_3^+) s^{-1}**				
**Average pH**	**272.6 K**	**278.0 K**	**283.5 K**	**292.5 K**
**1.39**	252.1±2.5	418.5±4.2	591.9±5.9	1204.9±12.1
**2.36**	223.8±2.2	366.7±3.7	542.0±5.4	1067.2±10.7
**2.70**	207.6±2.1	339.1±3.4	486.8±4.9	968.0±9.7
**3.17**	271.9±2.7	414.1±4.1	613.7±6.2	1257.5±12.6
**3.57**	377.1±3.8	575.9±5.8	889.5±8.9	1764.0±17.7
**3.86**	683.0±6.8	1052.0±10.6	1565.7±15.7	2921.2±29.5
**4.30**	3065.6±31.0	4107.9±41.8	5416.7±55.6	8397.4±88.6
**4.88**	17791.7±213.7	22581.6±296.7	27490.8±403.7	41970.8±939.3
**b) k_{ex} ($NH^{\varepsilon2}$) s^{-1}**				
**1.39**	1058.6±10.6	1529.5±15.4	2235.7±22.5	3998.0±40.6
**2.36**	1100.2±11.0	1623.3±16.3	2353.9±23.7	4142.2±42.1
**2.70**	1103.1±11.0	1648.6±16.6	2427.6±24.4	4218.7±42.9
**3.17**	1442.4±14.5	2119.9±21.3	3071.7±31.1	5377.7±55.0
**3.57**	1876.7±18.9	2786.9±28.1	4025.3±40.9	6980.8±72.1
**3.86**	3627.1±36.8	5131.4±52.4	7080.9±73.2	11562.0±123.3
**4.30**	12692±136.6	15758±173.9	19474.1±227.7	27363.7±347.7
**4.88**	68380.8±2725.1	84882.9±5365.4		
**c) k_{ex} ($NH^{\delta1}$) s^{-1}**				
**1.39**	11656.6±117.8	16099.1±163.5	22531.8±230.5	36719.8±388.6
**2.36**	12761.7±129.1	17909.5±182.2	24688.3±253.3	40844.1±440.8
**2.70**	12961.9±131.2	18316.4±186.5	25248.3±259.3	41681.8±451.7
**3.17**	13639.8±138.2	19072.6±194.3	26759.1±275.6	43864.4±481.7
**3.57**	14812.1±150.2	20631.6±210.6	28074.7±289.9	46728.2±523.9
**3.86**	16895.0±208.2	23141.6±236.9	31536.2±328.2	49126.8±561.3
**4.30**	32657.7±340.7	42538.6±457.0	52583.1±609.0	80903.8±1485.1
